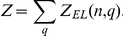# Computing the Partition Function for Kinetically Trapped RNA Secondary Structures

**DOI:** 10.1371/journal.pone.0016178

**Published:** 2011-01-28

**Authors:** William A. Lorenz, Peter Clote

**Affiliations:** 1 Department of Mathematics and Computer Science, Denison University, Granville, Ohio, United States of America; 2 Biology Department, Boston College, Chestnut Hill, Massachusetts, United States of America; University of North Carolina at Charlotte, United States of America

## Abstract

An RNA secondary structure is *locally optimal* if there is no lower energy structure that can be obtained by the addition or removal of a single base pair, where energy is defined according to the widely accepted Turner nearest neighbor model. Locally optimal structures form kinetic traps, since any evolution away from a locally optimal structure must involve energetically unfavorable folding steps. Here, we present a novel, efficient algorithm to compute the partition function over all locally optimal secondary structures of a given RNA sequence. Our software, RNAlocopt runs in 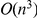 time and 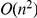 space. Additionally, RNAlocopt samples a user-specified number of structures from the Boltzmann subensemble of all locally optimal structures. We apply RNAlocopt to show that (1) the number of locally optimal structures is far fewer than the total number of structures – indeed, the number of locally optimal structures approximately equal to the square root of the number of all structures, (2) the structural diversity of this subensemble may be either similar to or quite different from the structural diversity of the entire Boltzmann ensemble, a situation that depends on the type of input RNA, (3) the (modified) maximum expected accuracy structure, computed by taking into account base pairing frequencies of locally optimal structures, is a more accurate prediction of the native structure than other current thermodynamics-based methods. The software RNAlocopt constitutes a technical breakthrough in our study of the folding landscape for RNA secondary structures. For the first time, locally optimal structures (kinetic traps in the Turner energy model) can be rapidly generated for long RNA sequences, previously impossible with methods that involved exhaustive enumeration. Use of locally optimal structure leads to state-of-the-art secondary structure prediction, as benchmarked against methods involving the computation of minimum free energy and of maximum expected accuracy. Web server and source code available at http://bioinformatics.bc.edu/clotelab/RNAlocopt/.

## Introduction

Kinetics of RNA secondary structure formation plays an important role in many biological functions, as shown by co-transcriptional folding [1] of large RNA molecules, the host-killing (hok) and suppression of killing system (sok) system [2] to control plasmid copy number in *E. coli*, the kinetically driven *trans*-splicing of a 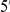 codon in *Leptomonas collosoma* [3], and the kinetic control in the formation of the *Tetrahymena* ribozyme [4].

RNA secondary structure kinetics depends on the distribution of *locally optimal* secondary structures, where a structure is said to be locally optimal if it is not the case that by adding or removing a single base pair, one can obtain a structure having lower free energy. In the context of the Nussinov energy model [5], where the energy of a base pair is 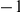, locally optimal structures are exactly the *saturated* secondary structures, as first defined by M. Zuker [6]. (A secondary structure is *saturated* if one cannot add any base pairs without violating the definition of a secondary structure; i.e. without either creating a base triple or pseudoknot.) In the paper [7] we developed an algorithm to compute the partition function for all saturated secondary structures of a given RNA sequence. Exploiting the idea behind this algorithm, in the papers [8], [9], we subsequently proved that the asymptotic number of saturated secondary structures is 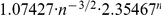, which (surprisingly) is not substantially less than the asymptotic number 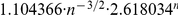 of all secondary structures, a result earlier proved by Stein and Waterman [10]. In Waldispühl and Clote [11], we extended our previous algorithm [7] to compute the partition function of all saturated secondary structures, with respect to the widely used Turner energy model [12]. In the Turner energy model, a secondary structure is decomposed into loops, as described in Zuker [13], and the free energy is computed by summing the energy contributions of all loops. A 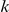-loop consists of 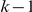 base pairs (excluding the closing base pair) and 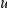 unpaired bases. The energies of 1-loops (hairpins), 2-loops (stacks if 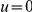, bulges or interior loops if 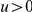), 3-loops and 4-loops (also known as 3-way and 4-way multiloop junctions) are obtained by least squares fit of enthalpy and free energy change at 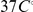, determined by optical melting (UV absorption) of small model systems [14], [15]. Even though free energies for the most common multiloops (3-way and 4-way junctions) have been experimentally determined [15], for computational efficiency it is usual to define the free energy of arbitrary multiloops (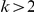) by the affine approximation 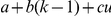, where 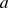, 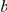 and 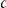 are constants.

Computational studies of RNA kinetics are currently performed either by repeated Monte-Carlo simulations, as in software Kinfold of Flamm, Fontana, Hofacker and Schuster [16], Kinefold of Xayaphoummine, Bucher, and Isambert [17], and RNAKinetics of Danilova, Pervouchine, Favorov, and Mironov [18], or by direct solution of the master equation from chemical kinetics 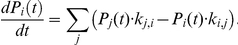



Here, 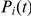 is the probability that the RNA molecule is in secondary structure 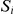 at time 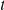, and 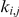 is the transitional probability of moving from structure 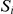 to neighboring structure 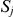, which differs from 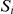 by the addition or removal of a single base pair, and where 
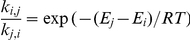
. By constructing probabilistic roadmaps for RNA secondary structure formation, a technique derived from robotic motion planning, Tang, Kirkpatrick, Thomas, Song and Amato [19] and Tang, Thomas, Tapia, Giedroc and Amato [20] are able to apply both Monte Carlo methods and the master equation over a smaller set of structures.

Flamm, Fontana, Hofacker and Schuster [16] describe RNA folding at an elementary step resolution, by using a Monte Carlo algorithm to study the kinetics of folding. Their Kinfold program is an implementation of Gillespie's Monte Carlo algorithm [21], [22] for stochastic folding, where elementary steps consist of either adding, removing or shifting a single base pair. In that paper, Flamm et al. describe the *barrier tree*, whose leaves are those locally optimal secondary structures having free energy that lies below a user-defined threshold. The barrier tree is constructed by using the program RNAsubopt [23] to exhaustively generate all secondary structures, whose free energy lies below a user-defined threshold, then aggregating structures into basins containing a locally optimal structure. As more structures are aggregated, using the imagery of *flooding* a landscape, two basins may be gradually joined together by folding paths, all of whose intermediate structures lie in one of the two basins, for which there exists a *saddle* structure of highest free energy along the path. Flamm, Hofacker, Stadler and Wolfinger [24] present additional applications of the Barriers program, while Wolfinger et al. [25] describe a coarse-grained approach by applying the *master equation* of chemical kinetics to macrostates consisting of basins of structures aggregated near locally optimal structures. For additional results on saddle points and energy barriers, see Stadler and Flamm [26], Flamm, Hofacker, Stadler, and Stadler [27], as well as the recent paper by Hofacker, Flamm, Heine, Wolfinger, Scheuermann et al [28], who introduce the notion of *barmap* which “links macrostates of temporally adjacent landscapes and defines the transfer of population densities from one ‘snapshot’ to the next”.

Other groups have studied various aspects of kinetically driven RNA folding. Shapiro, Bengali, Kasprzak and Wu [29] compute likely folding intermediates in the earlier described hok/sok system. Danilova, Pervouchine, Favorov, and Mironov [18] describe the web server, RNAKinetics, which models the secondary structure kinetics of an elongating RNA molecule. Xayaphoummine, Bucher, and Isambert [17] and Isambert [30] introduce the Kinefold web server, which stochastically folds a user-given RNA sequence into a low energy structure that may include pseudoknots. Quite recently, Dotu, Lorenz, Van Hentenryck and Clote [31] describe an efficient program RNAtabupath to compute near-optimal folding pathways between two secondary structures of a given RNA sequence. For an overview of RNA folding kinetics, see the review articles by Chen [32] and Al-Hashimi and Walter [1].

In this paper, we describe a novel, efficient algorithm, RNAlocopt, to compute the partition function over all secondary structures that are locally optimal in the Turner energy model. Locally optimal structures form *kinetic traps*, hence create *basins of attraction* in the energy landscape. The structure of this paper is as follows. In the introduction sections, we provide background definitions for the Turner energy model and loop decomposition. To allow the paper to be self-contained, we additionally describe McCaskill's classical algorithm for the partition function [33].

In the Results section, we present three types of analysis using the software RNAlocopt. First, by performing computational experiments on RNA sequences of increasing length, we show that the number of locally optimal structures is asymptotically the square root of the number of all structures, as depicted in Figure 5. Secondly, we compare the structural diversity, as measured by four different metrics, of the set of locally optimal structures with that of the Boltzmann ensemble of all secondary structures. Structural diversity appears to depend on the type of RNA; for instance, in the case of precursor microRNAs and 5S-rRNA, the structural diversity of the collection of locally optimal secondary structures is markedly lower than that over the Boltzmann ensemble, while structural diversity for TPP riboswitch aptamers appears to be about the same. Thirdly, we demonstrate how to combine McCaskill base pairing probabilities with those from sampled locally optimal structures in order to compute a modified *maximum expected accuracy* structure [34], [35], which appears to be closer to the native structure than structures produced by other thermodynamics-based algorithms. The Discussion section provides additional comments on the energy model of RNAlocopt and benchmarking issues, and as well describes intended future applications and possible extensions of the software. In particular, in forthcoming work, we will introduce a new method using RNAlocopt to quickly and accurately determine the *mean folding time* for a given RNA sequence, a synthetic biology application for *de novo* RNA design.

In the Methods section, we begin by describing the intuition behind the new 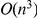 time and 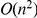 space algorithm, whose details and recurrence relations are then provided. Though our software RNAlocopt additionally can sample a user-specified number of structures from the Boltzmann subensemble of locally optimal structures, we do not describe details of the construction, since it is analogous to the construction of Ding and Lawrence [36], [37], albeit where the McCaskill partition function is replaced by the partition function for locally optimal structures.

### Background

**Figure 1:**
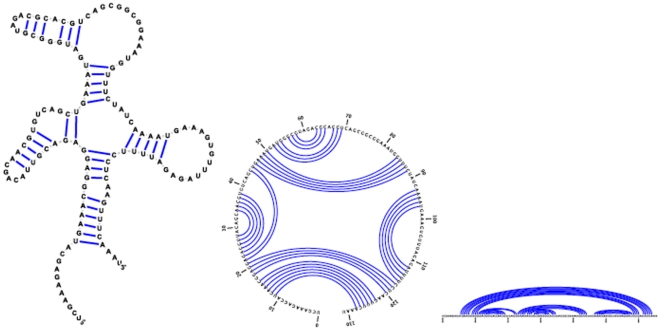
RNA from *human accelerated region* HAR1F, a region of the human genome that differs from highly conserved regions of our closest primate relatives and is active in the developing human brain between the 7th and 18th gestational weeks [38]. Secondary structure representation in conventional form (left), as a circular Feynman diagram (center) and as a linear Feynman diagram (right). Sequence and consensus secondary structure taken from Rfam [42]; graphics produced with jViz software [43].

**Figure 2:**
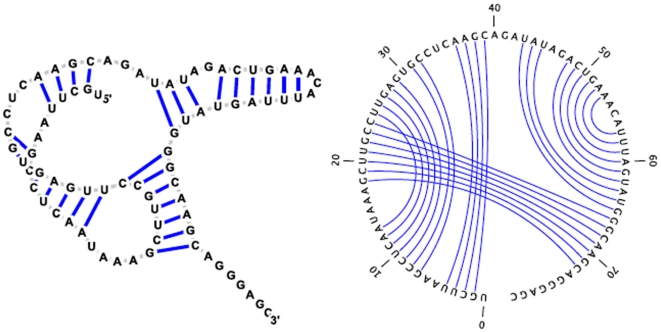
Long range pseudoknot PKB239 in the 5′ untranslated region (UTR) of human immunodeficiency virus HIV-1. Secondary structure with pseudoknots displayed in conventional form (left) and as a circular Feynman diagram (right). Sequence and structure of PKB239 taken from Pseudobase [44]; graphics produced with jViz software [43].

An RNA molecule is a biopolymer consisting of nucleotides, adenine (A), cytosine (C), guanine (G) and uracil (G), oriented in a natural left-to-right fashion given by the 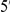 to 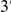 direction. Given an RNA sequence 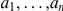 of length 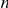, an RNA secondary structure 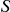 is defined to be a set of base pairs 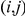, where *(a)* if 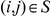, then 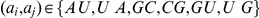 (base pairs are canonical, i.e. either Watson-Crick or wobble pairs); *(ii)* if 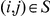, then 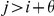, where by convention 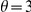 (minimum of 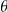 unpaired bases in a hairpin loop); *(iii)* if 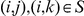, then 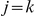 and if 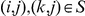, then 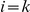 (non-existence of base triples); *(iv)* if 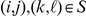, and 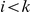, then 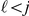 (non-existence of pseudoknots). See Figure 1 for three equivalent representations of the secondary structure for RNA from *human accelerated region* HAR1F, a region of the human genome that seems to have been under evolutionary pressure in the divergence of humans from great apes [38]. While secondary structures satisfy a planarity condition, pseudoknots violate that condition, as shown in Figure 2. Although pseudoknots and *non-canonical* base pairs play important roles in RNA tertiary structure formation [39], the secondary structure forms rapidly and serves largely as a scaffold for the formation of tertiary contacts [40]. In this paper, we are interested in developing an efficient algorithm to explore the energy landscape of kinetically trapped RNA structures. Since Lyngsø and Pedersen [41] have proved that it is NP-complete to compute the minimum free energy structure for a given RNA sequence, when general pseudoknots are permitted, we will restrict our attention throughfoout the paper to secondary structure.

### Nearest neighbor energy model

**Figure 3:**
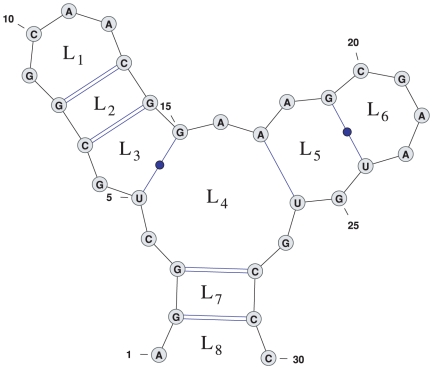
The Turner energy model is an additive *loop* model, whereby the free energy of an RNA secondary structure is defined to be the sum of loop free energies in a unique decomposition of the structure into loops. In this figure, the free energy of the depicted structure is the sum of free energies of loops 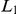 through 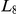. The Turner rules include free energy parameters for different types of loops, illustrated here for *hairpins* (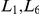), *stacked base pairs* (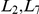), *bulges* (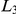), *internal loops* (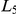), *multiloops* (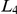) and *external loops* (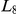). The Turner parameters are derived from a series of UV absorption (optical melting) experiments described in a number of papers including the references [12], [45]–[49]. For a complete list of all references, see http://rna.urmc.rochester.edu/NNDB/ref.html. Images created using the software VARNA [50].

The Turner nearest neighbor energy model is an additive model, where the free energy of an RNA secondary structure is computed as the sum of distinct loop free energies in a unique decomposition of the structure. Figure 3 illustrates the different types of possible loops for an example RNA secondary structure. The structure contains 8 loops of 4 basic different types. *Hairpins* are formed when a base pair 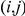 encloses an unpaired region of RNA; thus a hairpin contains the nucleotides 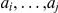, where due to steric constraints, 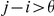, for 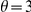, and positions 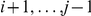 are unpaired. *Stacked base pairs* are loops containing adjacent base pairs, 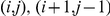, as shown in loops 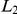 and 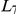. *Left bulges* are loops containing the closing base pairs 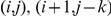 for 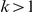, where 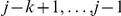 are unpaired; *right bulges* contain the closing base pairs 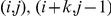 for 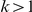, where positions 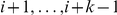 are unpaired. Loop 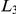 depicts a left bulge. *Internal loops* are loops bordered by 2 base pairs 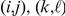, where 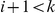 and 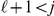. Loop 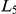 depicts an internal loop. A *multiloop* is a loop bordered by 3 or more base pairs. For instance, 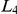 is a multiloop closed by the base pair 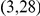, which here is a 3-way junction (i.e. bordered by three base pairs) and which has two *components* (i.e. stems bordered by base pairs 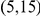 and 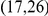). The number 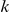 of base pairs that border a loop can be use to classify the loop; 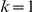 in hairpins, 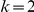 in stacked base pairs, bulges, and internal loops, and 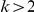 in multiloops. Finally, *external loops*, depicted in 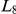, are technically not loops, but rather are defined to be regions containing nucleotide positions 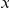 for which there is no base pair 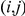 satisfying 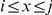.

In the Turner energy model, there are free energies for each type of loop. For the example structure 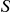 depicted in Figure 3, if we denote the energy of loop 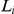 by 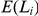, it follows that the free energy of 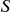 is
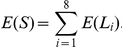



The Turner rules were fit to enthalpy and folding free energy change at 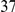
°C, determined by optical melting of small model systems [12], [51]. For instance, Turner's rules assign stacking free energy of 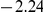 kcal/mol to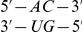 and of 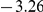 kcal/mol to 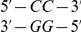. Stacked base pairs constitute negative (stabilizing) free energy contribution; hairpins, bulges, internal loops, and multiloops generally contribute positive (destabilizing) free energies, although certain 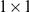 and 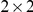 internal loops contribute stabilizing energies.

Important aspects of the Turner energy model are *additivity* and *locality*. Both of these properties are critical in the development of an efficient computation of the partition function; indeed, it is this local nature of the energy model that renders it possible to inductively determine all locally optimal structures.

The algorithm to compute the partition function of all locally optimal structures is a modification of McCaskill's algorithm, which we will review now. McCaskill's algorithm recursively computes the partition function for structures on subsequence 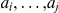 by table look-up of the previously computed partition function values for proper subwords of 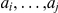. Each recursion step involves the addition of either one base pair or one unpaired base to groups of structures whose partition function is already known. Our modification to McCaskill's algorithm is to make sure at each step that the base pair or base added does not cause the occurence of non-optimal structures. This will require additional information to be stored at each step, but does not change the basic structure of the McCaskill recursions.

### McCaskill's partition function

In order to provide a self-contained treatment, we now review the construction of McCaskill's algorithm [33] to construct the partition function for RNA secondary structures.

Given RNA nucleotide sequence 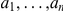, we let 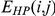 denote the free energy of a hairpin closed by base pair 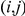, while 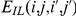 denotes the free energy of an *internal loop* enclosed by the base pairs 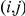 and 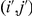, where 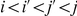. (Internal loops comprise the cases of stacked base pairs, left/right bulges and proper internal loops.) The free energy for a multiloop containing 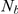 base pairs and 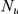 unpaired bases is given by the affine approximation 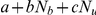.

Given an RNA sequence 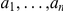, for 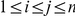, the McCaskill partition function 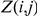 is defined by 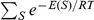, where the sum is taken over all secondary structures 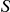 of 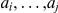, 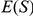 is the free energy of secondary structure 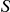, 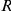 is the universal gas constant, and 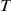 is absolute temperature. In the sequel we write 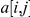 to abbreviate 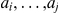.

### Definition 1 (McCaskill's partition function)

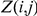
: partition function over all secondary structures of 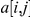.

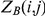
: partition function over all secondary structures of 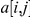, which contain the base pair 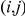.

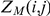
: partition function over all secondary structures of 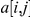, subject to the constraint that 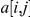 is part of a multiloop and has at least one component.

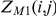
: partition function over all secondary structures of 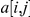, subject to the constraint that 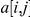 is part of a multiloop and has at exactly one component. Moreover, it is required that 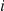 base-pair in the interval 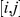; i.e. 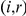 is a base pair, for some 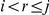.

Following McCaskill [33], the unconstrained partition function is defined by
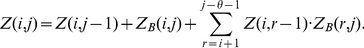
(1)


The constrained partition function closed by base pair 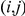 is given by
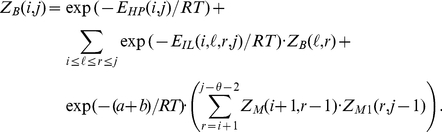
(2)


The multiloop partition function with a single component and where position 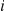 is required to base-pair in the interval 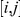 is given by 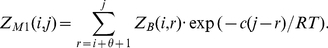
(3)


Finally, the multiloop partition function with one or more components, having no requirement that position 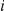 base-pair in the interval 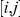 is given by
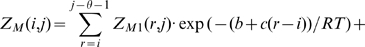
(4)

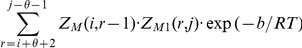



**Figure 4:**
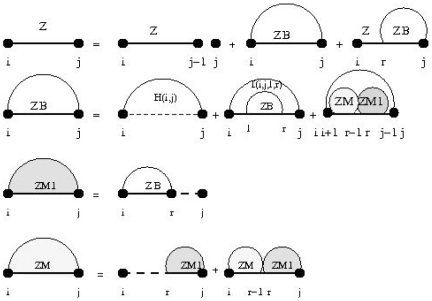
Feynman diagram of original recursions from McCaskill's algorithm [**33**] to compute the partition function. (Notation in this figure slightly deviates from that in text; e.g. 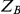 in text corresponds to 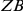 in the figure.)

See Figure 4 for a pictorial representation of the recursions of McCaskill's (original) algorithm [52]; note that the recursions are equivalent to, but not quite the same as, those given in [53].

## Results

### Number of locally optimal structures

In this section, we compare the values of the partition function, 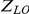, of all locally optimal structures, and the total number, 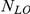, of locally optimal structures, with those for all structures. The number of locally optimal structures, 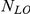, is determined by removing all energy factors in the previous equations for the Boltzmann partition function. This is equivalent to setting the temperature to 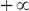, since all energetic factors are of the form 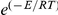.

**Figure 5:**
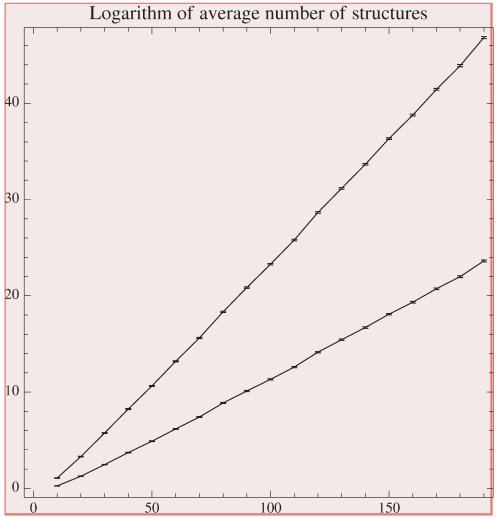
This figure depicts the logarithm (base 10) of the number of locally optimal [resp. all] secondary structures for random RNA. Sequence length is given on the 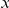-axis, while the logarithm of the number of locally optimal structures (lower curve) [resp. all structures (top curve)] is given on the 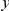-axis. Error bars are displayed. For various lengths 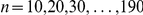, random RNA sequences of length 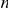 were generated by a 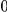
th order Markov process with probability 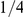 for each nucleotide A,C,G,U. For each value of 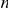, the average (exact) number of locally optimal [resp. all] secondary structures was computed. Using least-squares fitting, we find that the number 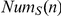 of secondary structures for length 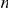 random RNA satisfies 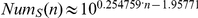 with 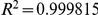, while the number 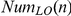 of locally optimal structures for length 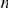 random RNA satisfies 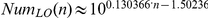 with 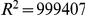. (The coefficient of determination, 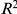, is the square of Pearson correlation coefficient of the least squares (linear) fit of the logarithm of the average number of structures.) It follows that the total number of structures is approximately equal to the number of local optima squared.

In Figure 5, for lengths between 20 and 200 nt, 100 RNA were randomly generated for each length in the simplest possible manner, with 1/4 probability of A, C, G, and U at each location. For each such RNA, the number of locally optimal structures as well as the number of all secondary structures is determined. These are averaged over the 100 randomly generated RNA sequences of that length, and plotted in the graph shown in Figure 5. We find there is exponential growth in the average, or expected, number of locally optimal structures, as a function of sequence length. Moreover, the slope of the curve in Figure 5 for the total number of structures is approximately twice that of the number of locally optimal structures, hence implying that the number of structures is approximately the square of that for locally optimal structures. Indeed, by fitting the data with a least-squares approximation, we find that the number 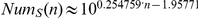 with 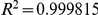, while the number 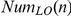 of locally optimalstructures for length 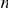 random RNA satisfies 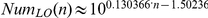 with 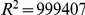. (The coefficient of determination, 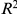, is the square of Pearson correlation coefficient of the least squares (linear) fit of the logarithm of the average number of structures.)

**Figure 6:**
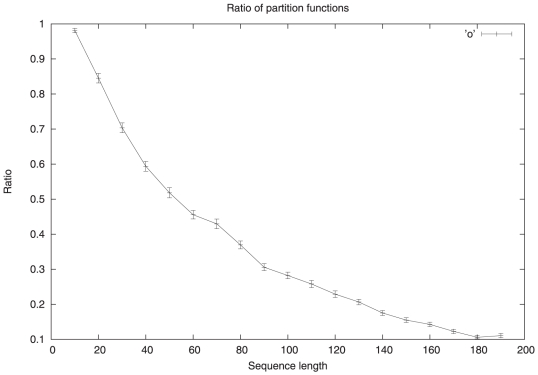
Plot of ratio, with error bars, of the restricted Boltzmann partition function 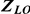 and the total Boltzmann partition function, as a function of RNA length, for the same random RNA generated as described in the **Figure 5**. This ratio represents the percentage of structures, as weighted by their Boltzmann factor, that are locally optimal. By numerical fitting, we find that this ratio is approximately 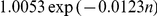 with coefficient of determination (see [54]) 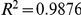.

In Figure 6, we compare the partition function, 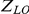, of all locally optimal structures, with the partition function, 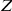, of all structures, by plotting the ratio, 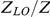, by the same method, averaging over 100 RNA at each length. This ratio, depicted with error bars, represents the percentage of structures, as weighted by their Boltzmann factor, that are locally optimal. By numerical fitting the data from this curve, it appears that the ratio is approximately 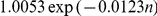 with coefficient of determination 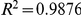 (see [54] for explanation of how to compute the coefficient of determination).

Another interesting computational experiment we performed was to determine the sum of the Boltzmann factors for a non-redundant subset of 1000 sampled locally optimal structures, produced by RNAlocopt, compared with the sum of the Boltzmann factors for a non-redundant subset of secondary structures, sampled by the Ding-Lawrence algorithm [36], as implemented in RNAsubopt -p. Table 1 presents these results for RNA generated in the previously described manner from an order 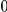 Markov chain, for lengths from 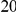 to 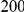 in steps of 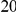. For each length, we averaged statistics over 10 runs, where for each run, we computed the percent coverage of the partition function; i.e. sum of the Boltzmann factors of a non-redundant subset from 1000 samples generated by RNAsubopt [resp. RNAlocopt], divided by the partition function 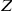 [resp. partition function 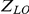 of locally optimal structures]. The number of locally optimal structures is far fewer than that of all structures (see Figure 5), hence, there is proportionately more redundancy among sampled locally optimal structures than than that over all structures. As well, the percentage coverage of the partition function for sampled locally optimal structures is higher than that for the Boltzmann ensemble.

**Table 1:** Using a 0th order Markov chain with probabilities of 0.25 for each nucleotide A,C,G,U, 50 random RNA sequences were generated for each length 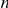, from 20 to 200 in steps of 20.

SeqLen	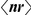 RNAsubopt	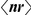 RNAlocopt	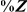 RNAsubopt	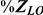 RNAlocopt
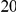	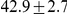	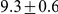	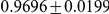	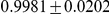
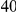	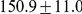	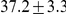	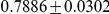	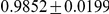
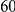	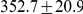	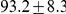	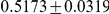	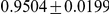
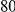	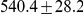	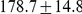	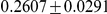	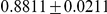
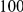	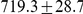	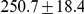	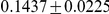	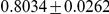
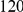	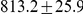	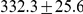	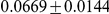	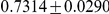
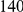	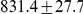	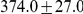	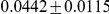	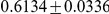
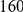	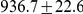	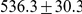	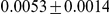	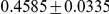
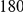	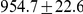	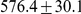	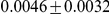	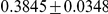
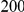	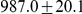	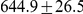	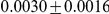	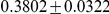

For each value of 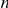, 1000 structures were sampled, by applying the Ding-Lawrence sampling algorithm [36], as implemented in RNAsubopt with flag -p, and by applying RNAlocopt. For each run, the number of non-redundant samples is computed, yielding the expected number 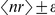 for RNAsubopt and RNAlocopt, where 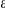 is the error bound (standard deviation 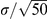, since 50 sequences generated). For each run the percent coverage of the partition function was computed; i.e. the sum of the Boltzmann factors of the non-redundant collection from 1000 samples generated by RNAsubopt [resp. RNAlocopt], divided by the partition function 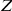 [resp. partition function 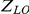 of locally optimal structures]. Since the number of locally optimal structures is far fewer than that of all structures (see Figure 5), it is not surprising that there is proportionately more redundancy among sampled locally optimal structures than than over all structures. As well, the percentage coverage of the partition function for sampled locally optimal structures is higher than that for the Boltzmann ensemble.

### Structural diversity of ensemble of locally optimal structures

In our paper on RNA saturated structures [55], we suggested that *(a)* there are far fewer locally optimal structures than there are of saturated structures, and *(b)* base pairing probabilities over locally optimal structures are similar to the base pair probabilities over all structures. In the previous section, we have shown that *(a)* holds; indeed, Figure 5 shows that the number of locally optimal structures is approximately the square root of the number of all structures, while the papers [7]–[9], [56] show that the number of saturated structures lies closer to that of all structures. While statement *(b)* holds in some cases, such as for purine riboswitch aptamers, in other cases, such as for precursor microRNAs and 5S-rRNA, it does not hold.

To numerically quantify how closely the ensemble of locally optimal structures resembles the Boltzmann ensemble of all structures, we consider four measures: the *pseudo-entropy* for base pairing probabilities, the *average entropy* for the base pairing probabilities, and two forms of *structural diversity*, the first due to Morgan and Higgs [57] and the second described in the Vienna RNA Package [58].

For a fixed RNA sequence 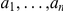 with base pairing probabilities 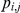, the *pseudo-entropy* is defined by 
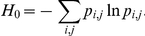



Since the collection of base pairing probabilities 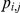 does not form a probability distribution (although it does for fixed 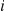, as exploited in the next definition), we cannot speak of its entropy, but rather use the term *pseudo-entropy*. The *average (Shannon) entropy* is defined by 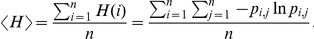



Both pseudo-entropy and the average entropy are measures of how well-defined are the base pairs. Indeed, if position 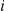 base-pairs with very different positions 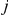 in the low energy ensemble of structures, then the entropy 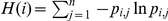 will be large. In contrast, if 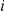 base-pairs with only one other position 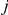, then 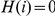.

The Morgan-Higgs structural diversity is defined by 
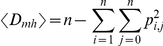
where 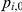 is defined by 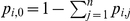. Finally, the Vienna structural diversity is defined by 
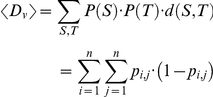
where the first sum is taken over all secondary structures 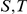 of a fixed RNA sequence, 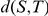 is the base pair distances between 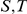, and 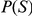 is the Boltzmann probability 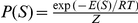 for structure 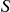 (and similarly for 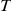). If there is no structural diversity whatsoever, so that 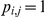 for all base pairs 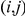 in the minimum free energy structure 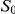, then clearly the Morgan-Higgs diversity 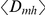 will take on the least possible value, 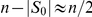, while the Vienna diversity will equal 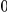.

Variants of the above measures are given as well for the ensemble of locally optimal secondary structures, where we use base pairing frequencies 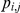 over a sampled collection of 1000 locally optimal structures for a given RNA sequence. Table 2 summarizes these four measures for 14 families of *seed alignments* from the Rfam 10.0 database [42]. For essentially all of these measures, we see that the structural diversity of the ensemble of locally optimal structures appears to be less than that for all structures. Notable exceptions are the riboswitch aptamers from Rfam.

**Table 2:** Structural diversity comparison between ensemble of locally optimal structures and Boltzmann ensemble of all structures.

Structural diversity								
Rfam family	L/M	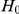	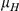	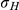	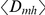	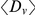	num	corrCoeff( 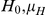 )
RF00001	L	14.403	0.247	0.231	23.276	15.160	710	0.6934
	M	19.122	0.327	0.283	28.543	18.918		
RF00003	L	29.167	0.357	0.285	42.046	29.487	100	0.70183
	M	36.703	0.450	0.33182	51.372	36.147		
RF00004	L	23.770	0.248	0.251	38.010	24.478	212	0.68387
	M	28.0856	0.294	0.295	42.623	27.779		
RF00005	L	11.637	0.315	0.259	18.013	11.811	1052	0.62225
	M	12.058	0.325	0.272	18.322	11.979		
RF00008	L	3.088	0.108	0.127	5.014	3.318	84	0.53836
	M	3.435	0.116	0.161	6.051	3.594		
RF00017	L	30.883	0.205	0.247	48.606	33.072	104	0.63080
	M	45.936	0.307	0.296	66.852	46.554		
RF00031	L	6.600	0.201	0.215	10.697	6.903	61	0.80180
	M	9.259	0.278	0.250	14.199	9.137		
RF00050	L	29.743	0.441	0.327	43.230	29.449	147	0.61214
	M	26.096	0.382	0.348	36.694	25.447		
RF00059	L	17.626	0.318	0.278	26.416	18.008	118	0.64729
	M	17.979	0.320	0.287	26.645	18.123		
RF00162	L	12.448	0.228	0.226	20.356	13.343	228	0.62482
	M	12.219	0.222	0.255	19.610	12.756		
RF00167	L	13.227	0.261	0.219	20.411	12.771	133	0.71049
	M	13.344	0.262	0.237	20.299	12.537		
RF00168	L	29.169	0.316	0.282	43.205	29.396	47	0.66707
	M	37.286	0.405	0.341	52.967	36.244		
RF00174	L	34.448	0.338	0.297	52.693	35.340	439	0.59984
	M	42.376	0.417	0.361	58.045	40.617		
RF00380	L	18.675	0.219	0.234	30.579	20.039	96	0.72220
	M	19.438	0.228	0.25112	30.801	20.266		

Given the collection of base pairing probabilities 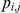 over all locally optimal structures [resp. over all structures] of a given RNA sequence 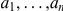, we define four measures of structural diversity. *(1)* The *pseudo-entropy* 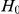 is defined by 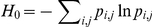. *(2)* The *average entropy* 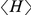 is defined by 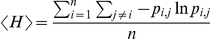. *(3)* The *Morgan-Higgs structural diversity* 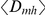 is defined by 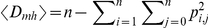, where we define 
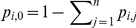
. *(4)* The *Vienna structural diversity* 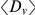 is defined by 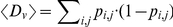. In the table above, we consider these measures with respect to locally optimal structures (L) and with respect to all (M) structures. (‘L’ stands for locally optimal, and ‘M’ for McCaskill.) The table depicts the number of structures for each Rfam family considered, as well as the correlation coefficient between pseudo-entropy and average entropy. The families in the table are: RF00001 (5S-rRNA), RF00003 (U1), RF00004 (U2), RF00005 (tRNA), RF00008 (hammerhead type III ribozyme), RF00017 (eukaryotic type signal recognition particle), RF00031 (selenocysteine insertion sequence), RF00050 (FMN riboswitch aptamer), RF00059 (TPP riboswitch aptamer), RF00162 (SAM riboswitch aptamer), RF00167 (purine riboswitch aptamer), RF00168 (lysine riboswitch aptamer), RF00174 (cobalamin riboswitch aptamer), and RF00380 (ykoK leader). Although we demonstrated a markedly lower structural diversity for locally optimal structures for precursor microRNAs, the data is not shown.

By using the new algorithm RNAlocopt, we have shown that the collection of locally optimal structures constitutes an ensemble that is *smaller* (see Figure 5) and structurally *less diverse* in general than that of all structures. This provides additional evidence for the hypothesis advanced in [24], [25], [27] that locally optimal structures form *basins of attraction* in the folding landscape of RNA secondary structures. For this reason, RNAlocopt may prove valuable in the study of kinetics of RNA folding.

### Basepair probabilities lead to better RNA secondary structure prediction

In ground-breaking work, Knudsen and Hein [59], followed by Do, Mahabhashyam, Brudno and Batzoglou [60] and by Kiryu, Kin and Asai [34], introduced the notion of *maximum expected accuracy* secondary structure, shown to be closer to the *native* structure, compared to the minimum free energy structure, when benchmarked against known structures. The underlying idea of this new approach is that there is a strong signal in the Boltzmann ensemble of low energy structures – a signal that is ignored when one computes the minimum free energy (MFE) structure, which is the *maximum likelihood structure* with respect to Boltzmann probability. Independently and at the same time, Ding, Chan and Lawrence [61] also realized the benefit of considering the Boltzmann ensemble rather than the MFE structure in their construction of the *Boltzmann centroid* of a cluster of sampled structures.

Following [34], [35], [59], [60], we define the *maximum expected accuracy* (MEA) structure for a given RNA sequence to be that which is obtained by tracebacks, using the matrix 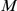, defined as follows:
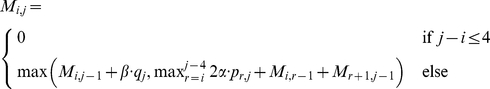
where 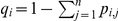, and 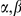 are non-negative constants. In the previous studies [34], [35], optimal values of 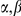 were found to be 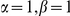. In this paper, we have set 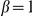 and performed benchmarking for a range of values 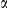 in 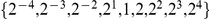. If most structures in the Boltzmann ensemble contain the base pair 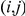, then 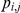 will be large, and it can happen that 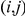 will belong to the MEA structure even though 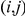 does not belong to the MFE structure. The values 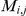 can be computed by a simple modification of the Nussinov-Jacobson algorithm [5], and the maximum expected accuracy structure with score 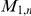 can be subsequently computed by tracebacks. See the references for more details, where the previously cited authors list benchmarking statistics to determine optimal parameters 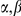 for which the MEA structure is closer to the native structure than is the MFE structure.

We compare variants of the MEA construction, obtained by using *(i)* base pairing probabilities 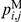 computed by McCaskill's algorithm [33] using RNAfold, *(ii)* base pairing probabilities 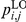 for locally optimal structures computed by relative frequency count from 10,000 sampled locally optimal structures, and *(iii)* base pairing probabilities 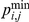, and unpaired probabilities 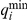, defined as the minimum of both probabilities; i.e. 
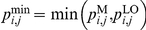
, and 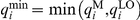. Note that in case *(iii)* it is no longer the case that 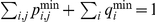. Cases *(i)*, *(ii)*, and *(iii)* yield the base pairing distributions 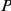 (McCaskill), 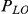 (locally optimal) and 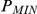 (minimum of McCaskill and locally optimal).

**Figure 7:**
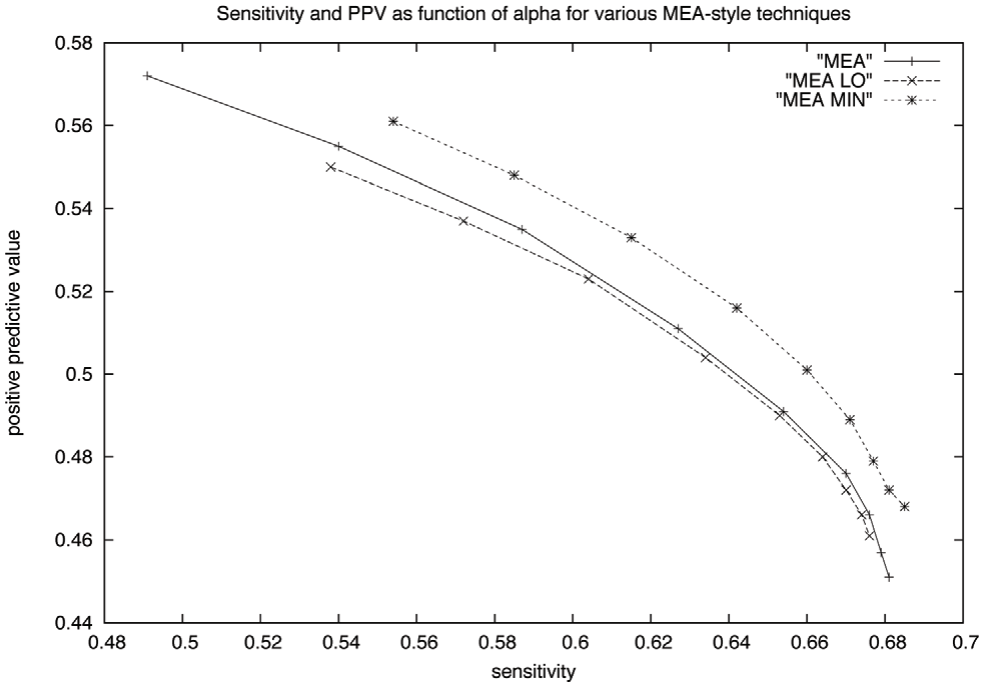
Graph showing *sensitivity* and *positive predictive value* for variants of the MEA method, when benchmarked with consensus structures from all *seed alignments* of Rfam 10.0 database [42]. For various values of 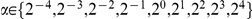 with 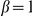, the sensitivity and PPV were computed for methods MEA, MEA LO and MEA MIN. Sensitivity of a secondary structure prediction for a given RNA sequence is defined as the number of correctly predicted base pairs divided by the number of base pairs in the native consensus structure, while PPV is defined as the number of correctly predicted base pairs divided by the number of base pairs in the predicted secondary structure. Sensitivity and PPV are computed by Rfam family, then averaged over all families of seed alignment in Rfam 10.0. (We performed a similar analysis where averages were taken over all sequences in Rfam, without first computing a family average. Results are similar; data not shown.) In [34], [35], the *maximum expected accuracy* (MEA) structure is computed by applying a variant of the Nussinov-Jacobson [5] algorithm using the base pairing probabilities 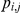 as computed by McCaskill's algorithm [33]. The parameter 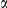 is a weight for base pairing probability; in other words, the *score*, following [34], [35], of a structure 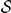 is given by 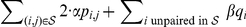. (Value 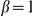 in the graph.) In the MEA LO variant of the MEA procedure, we consider base pairing frequencies 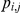, obtained by sampling locally optimal structures, while in the MEA MIN variant, we take 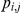 to be the minimum of the McCaskill base-pairing probability and the base pairing frequency sampled from locally optimal structures, *and* we take 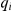 to be the minimum of the corresponding probabilities that 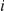 is unpaired in the low energy ensemble (using RNAfold -p) and in the locally optimal ensemble (using RNAlocopt). Sensitivity and PPV values are respectively 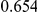 and 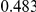 for the minimum free energy (MFE) structure, as computed by RNAfold from the Vienna RNA package [58], similar to the values for MEA, which latter has sensitivity 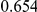 and PPV of 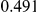 when 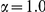. The single point below each of the three curves corresponds to MFE sensitivity and PPV. The method MEA MIN gives a consistent performance improvement over the other methods.

**Figure 8:**
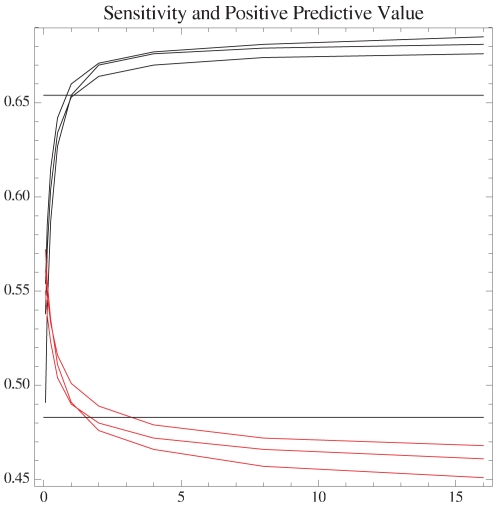
Graph showing *sensitivity* (black, increasing curves) and *positive predictive value* (PPV, red, decreasing curves) as a function of 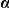 (explained in text and in Figure 7) for methods MEA, MEA LO, and MEA MIN. as benchmarked with consensus structures from all *seed alignments* of Rfam 10.0 database [42]. Values of 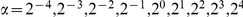 given on 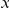-axis, while values of sensitivity and ppv are given on the 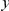-axis. Sensitivity and PPV are computed by Rfam family, then averaged over all families of seed alignment in Rfam 10.0. (We performed a similar analysis where averages were taken over all sequences in Rfam, without first computing a family average. Results are similar; data not shown.) The MEA MIN method yields a consistent improvement other MEA methods, as well as over minimum free energy (MFE) structure predictions, benchmarked by using RNAfold from the Vienna RNA package [58]. The best sensitivity and the best PPV are given by method MEA MIN; the next best by MEA LO, and the last by method MEA. Two horizontal lines indicate the sensitivity (top line) and PPV (bottom line) for the minimum free energy structure, as computed by RNAfold from the Vienna RNA Package.

We can determine corresponding MEA structures, denoted by 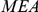 and 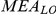, according to the use of 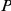 resp. 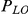. We see in Figures 7 and 8 that predictions based on these MEA structures are better than the MFE structure, as predicted by RNAfold. However the predictions based on local optima are consistently worse.

However, we can create a third matrix, denoted by 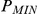, where for each base pair 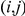, 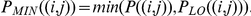



This will in essence emphasize those base pairs that occur prominently in both samples of local optima and samples of all structures. As shown in Figure 7, this consistently increases the sensitivity and positive predictive value.

## Discussion

In this paper, we describe a novel and efficient algorithm to compute the partition function over all locally optimal secondary structures of a given RNA sequence. The software, RNAlocopt runs in 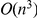 time and 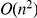 space, the same time and space complexity as that of McCaskill's algorithm to compute the partition function over all secondary structures. Additionally, RNAlocopt samples a user-specified number of structures from the Boltzmann subensemble of all locally optimal structures. Our work completely solves a line of investigation begun originally by M. Zuker [6], who first defined the notion of *saturated* structure (for which no base pair can be added without violating the definition of secondary structure).

The energy model implemented in RNAlocopt is the Turner nearest neighbor energy model *without* dangles; in contrast, the energy model used in the software RNAfold and RNAsubopt is the Turner model *with* dangles. Our computation of sensitivity and positive predictive value (PPV) is *exact*; i.e. with no allowed *slippage*. In contrast, some authors, such as Lu and Mathews [35], benchmark sensitivity and positive predictive values by allowing a *slippage* of 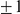; i.e. if base pair 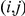 belongs to the native structure, then the predicted base pair 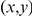 is counted as correctly predicted if 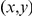 is one of the following: 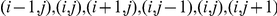. In [35], sensitivity and PPV values are reported with slippage for the maximum expected accuracy (MEA) method using the software RNAstructure [62], which includes energy terms for *coaxial stacking*.

There may be some discrepancies between reported sensitivity and PPV values from various groups. Such discrepancies will occur due to a combination of benchmarking with respect to different databases, admitting slippage or not, and small differences in the underlying energy model. Nevertheless, there is a consistent improvement of MEA MIN, as shown in this paper, over both minimum free energy (MFE) and maximum expected accuracy (MEA) methods.

By applying RNAlocopt to randomly generated RNA, we have shown that there are far fewer locally optimal structures than that of all structures (the number of locally optimal structures approximately equals the square root of the number of all structures). We have shown that the structural diversity, as measured by four different parameters, of samples of locally optimal structures can either be similar or quite distinct from samples from the Boltzmann ensemble of all structures – a situation that depends on the particular RNA family. While most RNA families we investigated displayed smaller locally optimal diversity than total structure diversity, notable exceptions were the riboswitch aptamers from Rfam. One might think that this is due to the fact that two distinct low energy conformations (gene-on and gene-off) are present in both the local optimal and Boltzmann ensemble. However, the Rfam database contains only the riboswitch aptamers, which do not undergo any significant conformation change. (Indeed, the riboswitch portion that undergoes conformation change, called the *expression platform*, is essentially missing from the Rfam data, a situation we will address in a future publication.) Thus it remains unclear exactly why riboswitch aptamers should display a difference in structural diversity between locally optimal and all structures.

Since there are relatively few locally optimal structures, compared to all structures, we are led to the hypothesis that in certain circumstances, a collection of sampled locally optimal structures can more succinctly represent the folding landscape of a given RNA sequence. In forthcoming work, we will describe an application of this observation, by presenting a new method for *de novo* RNA structure design, where kinetic properties are taken into account.

Theoretical studies of RNA folding kinetics have primarily focused on *unit-step* resolution, where a single base pair is added or removed in each time step. For such studies, RNAlocopt will prove to be a valuable new tool. There is some possibility of extending RNAlocopt to allow the formation or removal of entire helices in each time step, a direction we are currently considering. The idea would be to redefine a *locally optimal* structure to be one for which no addition or removal of any stem region would lower the free energy. An extension of RNAlocopt in this direction would allow more rapid exploration of the folding process.

Locally optimal structures 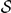 form kinetic traps, in the sense that there does not exist a structure 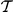, obtained from 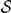 by the removal or addition of a single base pair, which has lower free energy. Since thermal noise can overcome the energy barrier between certain conformations in the low energy ensemble, a better model of kinetic trap might arguably be a that of a basin of attraction located about locally optimal structure 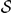. Such a basin would be a *set* 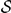 of low energy structures, such that: *(i)* there is a folding path whose barrier energy is less than a fixed energy threshold 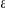 that cannot be overcome by thermal noise, and *(ii)* if 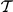 is reachable by a folding pathway from 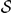 with barrier energy less than 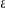, then 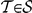. Though it is currently unclear what value of 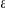 should be taken, it may be possible to extend RNAlocopt in this direction. This is a possible avenue for future research. (A folding pathway from 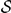 to 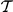 is a sequence 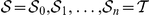 of secondary structures, such that 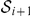 is obtained by adding or removing a single base pair from 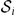, for each 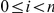. The barrier energy of a folding pathway is 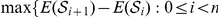. Computing *optimal* folding pathways between any two secondary structures is known to be NP-complete, though there are exponential time *exact* algorithms [24], [63] and efficient *near optimal* algorithms [31].)

Finally, we have shown the utility of locally optimal structures by demonstrating that the variant of maximum expected accuracy structure, MEA MIN, provides the most accuracy structure prediction currently available via thermodynamic methods. The improvement in sensitivity and PPV for this method depends on the fact that we take into account the base pairing frequency of pairs 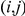 within the ensemble of locally optimal structures as well as that of the Boltzmann ensemble of all structures.

Why is the MEA MIN structure apparently closer to the native structure, at least in the benchmarking study performed in this paper? Since there is no clear answer to this question, we can only formulate a guess. Recall that there are far fewer locally optimal structures than there are of all secondary structures, and that the ensemble of locally optimal structures appears to be more consistent (i.e. less structurally diverse, at least in most cases) than the ensemble of all structures. For these two reasons, certain unlikely, pathological candidate base pairs have diminuished likelihood of contributing to the MEA LO structure. However, certain important intermediate structures, which do not appear in the ensemble of locally optimal structures, could contribute to the accuracy of the MEA structure. By taking the minimum of base pairing probabilities over both ensembles, MEA MIN is closer to the native structure. Though reasonable, we must stress that this explanation can only be speculative.

## Methods

We begin by providing an intuitive overview of the construction, while subsequent sections provide full details and the recurrence relations for the RNAlocopt algorithm.

### Conditional local optimality

To implement our algorithm, at each step we wish to calculate the partition function of only the locally optimal structures. Since the Turner energy model is a loop-based model, it can largely be construed as a local model. Therefore we can locally check whether or not adding the base pair 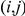 makes some structures suddenly no longer locally optimal simply by looking at nucleotides near 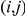. To do this, we need to keep track of a bit more information during our recursion than is done in McCaskill's algorithm.

**Figure 9:**
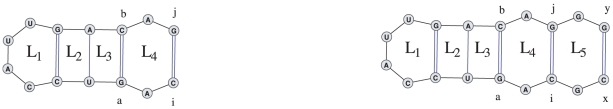
Example structure in recursion. In the left structure, we do not yet know the two loops bordered by the base pair 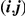. Therefore we do not yet know whether by removing this base pair, the free energy will be lowered. In the right structure, one step further in the recursion, we now know which loops border the base pair 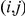 – namely, loops 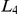 and 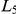. Images created using the software VARNA [50].

In this section we show through a simple example the key idea behind the recursions. Consider the partial sequence-structure shown in the left side of Figure 9. The Boltzmann factor (portion of the Boltzmann partition function) of this structure would be included in the term 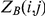 in McCaskill's recursion, which denotes the partition function of all structures ending in a base pair at 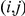.

It would be natural to define an analogous term 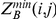 as the partition function of all locally optimal structures ending in a base pair at 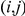. Local optimality would mean that adding or removing any base pair would raise the energy, or keep it the same. But in our example structure, there is one base pair for which we do not have sufficient information to know the change in energy caused by removing it – namely, the outer base pair 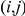. In the next recursive steps, this structure could be extended in several different possible ways, perhaps with a base pair 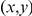 shown in the right-hand side of Figure 9. At that point, we will know the energy of the two loops in which the base pair 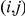 is contained. But until then, this energy is unknown.

Since we do not yet know how removing the base pair 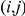 will affect the energy, the best we can do is to inductively assume that the structure is conditionally locally optimal, conditioned on the fact that 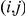 must base pair. It will not be until we add the next base pair 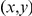 that we will know whether the base pair 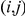 causes the structure to not be locally optimal, that is if removing the base pair 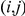 decreases the energy.

**Figure 10:**
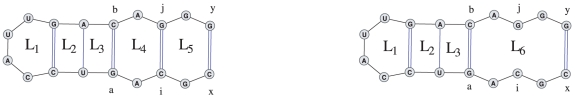
Example structure in recursion. The energy change effected by removing the base pair 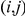 is 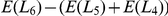. To calculate this, we need to keep track of base pair 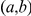. Images created using the software VARNA [50].

Consider then the structure including the base pair 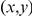 on the left side of Figure 10. Remember that we could not determine the change in energy caused by removing base pair 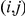 before. That change in energy is now given by the energy of the new loop, 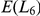 minus the energy of the old loops, 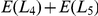, as indexed in Figure 10. However, to determine the energies 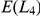 and 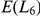, we need to know the location of the base pair 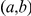.

Our approach for this internal loop example is to induct on the last two base pairs, not just the last base pair. So in our example, our example structure on the left-hand side of Figure 9 will contribute to the term 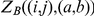, which denotes the partition function of all locally optimal structures with the outermost two base pairs 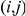 and 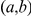. Then, if removing the base pair 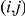 doesn't lower the energy, that is if 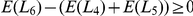, the structure on the right-hand side of Figure 9 will contribute to the term 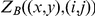.

We must also check if any base pairs can be added. In our example, when adding the base pair 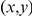, we check if any base pairs can be added within the internal loop 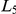 defined by 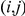 and 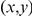 (see Figures 9 and 10). Any other base pair additions would already have been considered earlier in the recursion, and the energy change of adding different base pairs is independent due to the loop energy model.

The previous discussion deals with internal loops. For external loops and multiloops, the motivation is similar, but the approach is more difficult, and the solution, which is more time-consuming and depends at least theoretically on the parameters of the Turner energy model, is less satisfying. As the recursion progresses, the conditionality of the optimality will be pushed outward, and in checking the final external loop, the conditionality will be removed, giving the full partition function and completing the recursion.

### Details of recursion for locally optimal structures

To do our recursion, we need to know the energies of various internal loops, hairpins, and the energies associated with a multiloop in the Turner energy model. These are available as temperature-dependent parameters. For simplicity, all calculations will be at 37°C.

We let 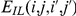 denote the free energy of an internal loop enclosed by two base pairs, 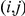 and 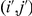, where 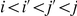. The energy of a hairpin enclosed by a base pair 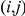 will be denoted by 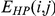. For a multiloop, such notation is not possible. The accepted energy of a multiloop is given by a multiloop penalty, 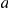, a penalty for unpaired bases in a multiloops, 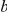, and a penalty or bonus for a base pair within a multiloop, 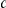, which can depend on the type of base pair being considered. The energy of the multiloop is then 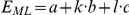
where 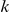 is the number of unpaired bases in the multiloop, and 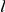 is the number of bases in the multiloop. This is standard, as used in McCaskill's algorithm, and is done in part for computational reasons. There is no affine energy term associated with external loops, but their treatment is somewhat analogous to that of multiloops (indeed, a multiloop can be formed by adding a closing base pair to an external loop).

### Explanation of deltas

The method of calculating local optima is straightforward. We will calculate the partition function of locally optimal structures with the same basic McCaskill algorithm used to calculate the partition function over all secondary structures. However, some modifications must be made, for at each step in our recursion, we must make sure that no base pair can be added or removed that would lower the energy. Anything that does not satisfy this property is dropped from the partition function.

**Figure 11:**
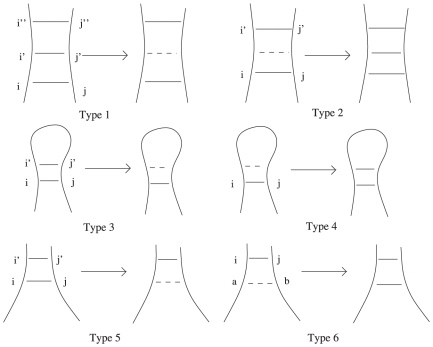
The six ways that a single base pair can be added to or removed from a structure and possibly reduce the overall energy. Images created using the software VARNA [50].

The way this is done is to realize all of the different ways a single base pair can be added and removed that can lower the total energy, and to build in a check for all of these cases as we build the partition function. Figure 11 shows all of the possibilities. A base pair can be added to or removed from a hairpin, (Types 3 and 4), an internal loop, which includes bulges and stacked base pairs (Types 1 and 2), or a multiloop (types 5 and 6).

In our recursion, we will have six different delta functions corresponding to these six different cases, where each delta function is 1 if adding, or removing, the relevant base pair does not lower the energy. Such deltas will act as checks whether the structures built so far are locally optimal.

For example, to check whether we can remove a base pair from between two internal loops, we have, from type 1 in Figure 11, 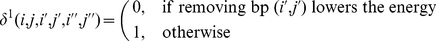



This delta is calculated using the energies of a given segment. The energy of the internal loops before removing the base pair are 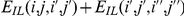
and after removing the base pair, the energy of the resultant single internal loop is 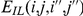



Thus we calculate delta by the formula 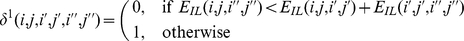



Other deltas are computed in a similar fashion. For types 2 and 4 (in Figure 11), these are precomputed, in order to speed up the algorithm. This precomputation gives us a list (each of order 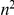 for a sequence of length 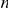) of possible IL's and HP's respectively, to which an internal base pair cannot be added which would lower the energy.

**Figure 12:**
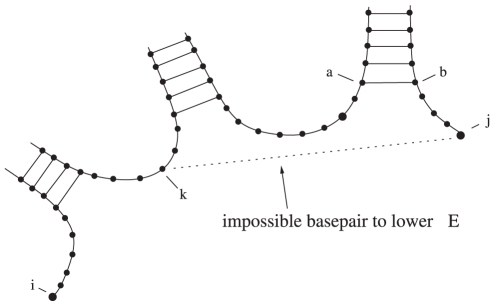
Image of base pair that could not possibly lower the energy by creating a multiloop, since it creates two bordering multiloops. Images created using the software VARNA [50].

One note is that some base pairs are never favorable, and thus do not need to be calculated. The important case is adding a base pair to a multiloop, which would split the multiloop into two multiloops when the multiloop is closed. This type of base pair is shown in Figure 12. Provided that there are no energy terms for either dangles within a multiloop, or coaxial stacking, this base pair will never lower the energy. This is fortunate, since it is computationally more difficult to inductively include such base pairs.

### Tails, conditional optimality

Just as there are hairpins, internal loops, multiloops, and external loops in the Turner energy model, there are recursion terms for hairpins, internal loops, multiloops, and external loops. However, as we need to keep a little more context to keep track of whether we still have a set of local optima, there will be some extra information.

Note that all these structures will be conditionally locally optimal. We commonly can't know if the most exterior base pair will be locally optimal, as that will depend on future base pairs, thus we need this conditional optimality in order to perform the recursion.

For example, for internal loops, we will denote 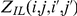
for the partition function of all locally optimal structures on the subinterval 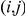 with an internal loop with base pairs 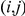 and 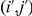, where 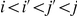. This local optimality is conditional on 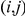 being a base pair, that is, we assume 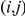 is a base pair, and will check later if this is a problem. We cannot tell whether, in the future, removing the base pair 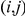 will lower the energy or not, as we don't know the structure outside of 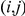.

**Figure 13:**
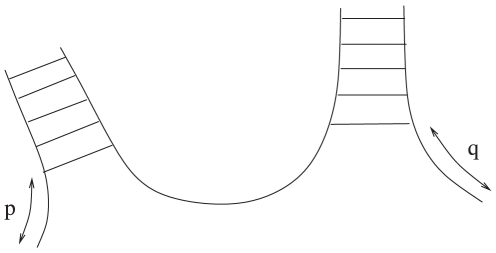
Example of the formation of a multiloop with tails of length 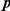 and 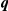. Images created using the software VARNA [50].

A few of the recursive elements will contain tails. For example, for multiloops, we will let 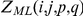
denote the partition function, on the interval 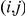, for all unclosed locally optimal multiloops (with more than one base pair) that have ‘tails’, regions of unpaired nucleotides, of lengths 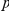 and 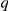 on the left and right side respectively. See Figure 13.

These tails are needed. In McCaskill's algorithm, for a multiloop closed by base pair 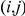, there is a recursion of the form 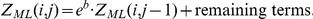



**Figure 14:**
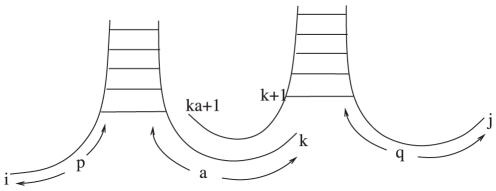
Example of gluing together two pieces of a multiloop. Note that if each piece is locally optimal, then the composite, obtained by gluing the pieces together, is as well. Images created using the software VARNA [50].

We cannot use such a recursion, as adding an unpaired base may result in a structure that is no longer a local optimum. While there may be better approaches, we avoid this problem by indexing locally optimal multiloops by their tail length. We can then glue such multiloops together with tails. See Figure 14.

We have seen that 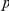 and 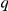 can be always less than 10, this is sufficient to avoid all possible base pairs in multiloops that lower energy. Almost all such base pairs can be avoided by setting 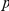 and 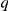 to be always less than 4; this allows for considerable speed-up with little loss of accuracy.

Note that we need the assumption that a single base pair cannot split a multiloop into two multiloops and thereby lower the energy. (This is true under the present Turner energy model. See Figure 12.) Otherwise, such a gluing method could result in a base pair being possible that lowers the energy – that is, the structure would not be locally optimal.

### Recursion Relations

Let 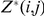 denote the partition function of all structures ending in the base pair 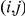 which will enter a multiloop. Note that we know from the Turner energy parameters that only an internal loop can enter a multiloop. It follows that 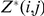 will be the sum of all possible internal loops ending in 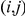. 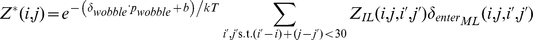
where 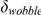 is 1 if we have an AU or GU base pair, 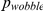 is the corresponding energy penalty, 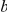 is the penalty of adding a base pair in a multiloop, and 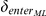 is 0 if removing the base pair 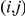 (and exposing the base pair 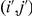 to the multiloop) lowers the energy. Thus (by induction) 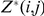 is the partition function for all structures that are locally optimal with respect to all of their base pairs, including 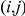.


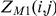
 is the partition function for locally optimal multiloops closed by base pair 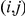 and having with exactly one component, while 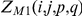 is the partition function for locally optimal multiloops with exactly one component, and which contains tails of length 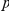 and 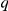. We let 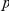, 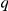 range from 0 to 10, with one extra position, called “>
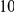
”, which is reserved for long tails. Thus 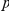 and 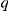 each have 12 possible values. (However, in practice, most values of 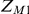 are not stored, but calculated as needed. Only those with 1 or 2 long tails need to be stored.)

The partition function 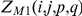 corresponds to having a base pair at 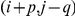 entering a multiloop, with tails out to 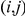, 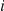 and 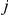 not base-paired. 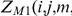
>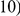 means an 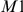 element with large right tail, greater than 10. This is used because if either tail is of length 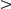
10, there are no longer any base pairs that can be added that can reduce the energy. This was shown by exhaustive search. This allows for traditional induction, since we don't have to worry about adding a base pair causing a formally locally optimal structure to become not locally optimal.

For 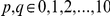, 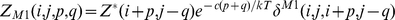


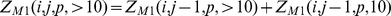


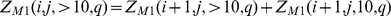


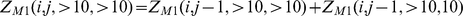
where 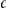 is the energy penalty of an unpaired base in a multiloop, and 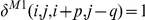 iff the base pair 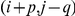 is such that no base pair 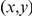 can be added 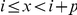, 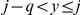, that lowers the energy. That is, the base pair 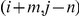 is locally optimal with tails in the multiloop of length 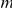 and 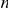 on the left and right respectively.

Note, we need another variable, 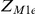, for the partition function of external loops with exactly one element. The recursion relations are almost identical. The only change is there is no base pair penalty.


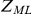
 is the partition function of multiloops with at least 2 exiting base pairs. Tails are glued together as in Figure 14. Notation is similar to 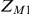, for the same reasons. Here, the recursion is quite nice.

Define the set 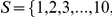
>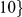. For 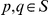


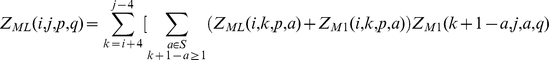
where in the expression 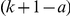, we replace 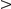
10 with 10. (This corresponds to unambiguously gluing the largest possible fixed tail. Otherwise there are several ambiguous ways to glue two long tails together.)

Thus we add a single exiting base pair with tails during the recursion. Note, with 12 possible tail lengths, the memory usage here is 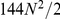. As cases of isolated base pairs far into a multiloop lowering the energy are rare, we can reduce the number of tail lengths recorded.

A similar equation for the external loop can be determined. Here we can always assume that the left end of the external loop (usually denoted with the variable 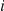) is 1, since we never need to close an external loop. Also, we need only worry about the right tail, for the same reason. Remember, an external loop can contain 0, 1, or more entering base pairs, corresponding respectively to the empty structure, structure with one component, and structures with more than one component. In this way it is slightly different than a multiloop.
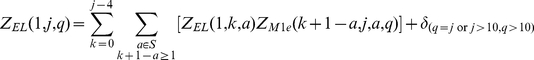
where 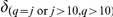 is 1 if 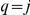 or (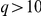 and 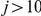), and where again in the expression 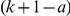, 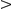
10 is replaced by 10. The term 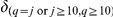 actually represents the empty structure. Note that 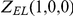 will be set to 1 by the above equation, as will 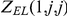. These can be thought of as representing the empty structure, or equivalently as initial conditions.

The variable 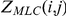 represents the partition function of all locally optimal closed multiloops ending in base pair 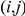. It is given by all of the ways to end a multiloop.
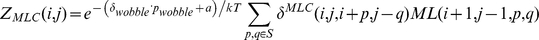
where 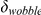, 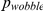 are as before, 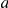 is the closing penalty of a multiloop, and 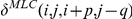 is 1 if there is no base pair 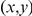, 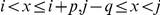, that would lower the energy of the multiloop. That is, within the available tails that close the ML, there is no way to add a base pair connecting these tails and lowering the energy.

All that is left is the partition function 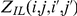. This is the partition function of all structures that are locally optimal, conditional on 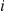,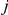 base-pairing, that exit in an internal loop with outermost base pairs 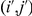 and 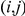, 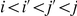. Following standard convention, we consider only internal loops of size at most 30; i.e. we can restrict to the case 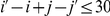.

There are 3 cases: *(i)* the internal loop borders a hairpin at 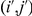, *(ii)* the internal loop borders a multiloop at 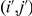, *(iii)* the internal loop borders another internal loop with base pairs 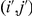, 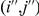. In all 3 cases, we need to do our inductive checks on optimality. For the last case, we must sum over all possible internal loops. (In practice, there is a prerecorded set of possible internal loops, increasing speed considerably.) The recursion is a sum over these three cases and is given by
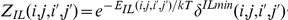


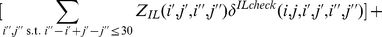


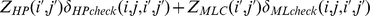
where 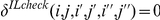 if removing the base pair 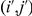 lowers the energy, and 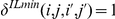 if no base pair 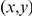, 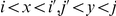 can be added that will (split the multiloop in two and) lower the energy. 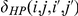 and 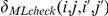 both check if removing 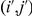 lowers the energy.


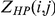
 is the partition function of a locally optimal hairpin with outer base pair 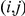, conditional on 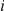, 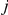 being base paired. We have
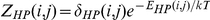
where 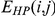 is the Turner energy for the hairpin with external base pair 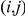, and 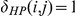 if the hairpin is locally optimal, that is if no base pair 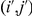, 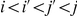, can be added that would lower the energy.

This gives consistent recursions. To calculate the total partition function, simply sum up all of the external loops with different tail lengths to yield